# Linking the NETSARC+ National Sarcoma Database With the SNDS to Evaluate Adjuvant and/or Neoadjuvant Therapy: Report on the Linkage Process and Result (Health Data Hub's DEEPSARC Pilot Project)

**DOI:** 10.1111/fcp.70066

**Published:** 2025-12-11

**Authors:** Erwan Drezen, André Happe, Vincent Thevenet, Nicolas Penel, François Gouin, François Le Loarer, Gonzague Du Bouexic De Pinieux, Hugo Crochet, Claire Chemin Airiau, Françoise Ducimetiere, Simone Mathoulin Pelissier, Jean‐Yves Blay, Emmanuel Oger

**Affiliations:** ^1^ CUBR SAS Rennes France; ^2^ University of Rennes Rennes France; ^3^ INSERM, Institut Bergonié, CIC 1401, Euclid/F‐CRIN Clinical Trials Platform University of Bordeaux Bordeaux France; ^4^ Medical Oncology Department Oscar Lambret Cancer Center and Lille University Lille France; ^5^ Centre Léon Bérard University Claude Bernard Lyon I Lyon France; ^6^ Institut Bergonié University of Bordeaux Bordeaux France; ^7^ CHU Tours University of Tours Tours France; ^8^ INSERM, Institut Bergonié, CIC 1401, Bordeaux Population Health U1219 Epicene Team University of Bordeaux Bordeaux France; ^9^ Department of Medicine Léon Bérard Center Lyon France

**Keywords:** data linkage, health insurance reimbursement, sarcoma

## Abstract

**Background:**

DEEPSARC, one of the first projects running on the Health Data Hub, aimed to identify real‐life treatment regimens that could improve overall survival. The project is based on matching the national database of the sarcoma reference network with the SNDS.

**Objectives:**

We aimed to report a transparent description of the linking process and its results.

**Methods:**

The sarcoma database encompasses 33 548 patients matching the selection criteria divided into three subsets: 13507 patients with a complete dataset gathering clinical and pathological data; 5844 patients with clinical data alone; and 14 197 patients with pathological data alone. As no ICD‐10 code reliably identifies patients with sarcoma, the subpopulation extracted from the SNDS was extended to 3 million patients who underwent surgery for their cancer. An indirect record linkage process used a combination (called a signature) of so‐called chaining variables to uniquely identify a pair of patients from each of the bases. Two metrics (signature robustness and overall quality) were calculated for ease of interpretation.

**Results:**

The overall matching rate of 73.1% (24 539 pairs out of 33 548 observations), reaching 90.5% in the intersection of the sarcomas databases (with extended data, 12 225 pairs out of 13 507 observations).

**Conclusion:**

An optimized and transparent process led to a moderate matching rate but enhanced the confidence in true matching. Representativeness is an issue related to the missing data pattern across the three NETSARC databases. For instance, an individual present only in the RREPS database has a greater probability of not being linked.

**Abbreviations**
CepiDC: Nationwide database containing data on causes of death derived from the treatment of certificates of death
CNIL: National Commission for Information Technology and Civil Liberties (French data protection authority)
HDH: “Plateforme des données de Santé” Health Data Hub
ICD: International Classification of Disease
MDTM: Multidisciplinary team meeting
NETSARC+: Sarcoma reference network since 2020 (merger of the three preexisting sarcoma networks since 2010): RRePS (pathological double review of soft tissue/visceral sarcoma)
(bone sarcoma): NetSarc (clinical management of soft tissue/visceral sarcoma) and ResOs
PMSI: Nationwide database containing data derived from the healthcare activity delivered in private and public establishments
SNDS: “*Système National des Données de Santé*” (French National Health Data System)
SNIIRAM: Nationwide database containing health insurance data derived from the processing of healthcare reimbursements, mainly for ambulatory care

## 1 Introduction

Although clinical data sources (clinical trials, epidemiological cohorts, or registries) provide valuable insights, even the best of them are limited by factors such as participant bias, attrition, and data inconsistency. These clinical data sources make it possible to collect the most relevant data for the field of research under consideration. For sarcomas, the multiplicity of histological types and subtypes, difficult to classify, leads to diagnostic errors [1] and a centralized double reading corrects up to 30% of diagnoses with a major impact on the choice of treatment [2, 3]. The national database of the sarcoma reference network (Sarcoma‐BCB) has recorded around 90% of patients with sarcoma since 2010, with centralized review in the 25 expert centers of the NETSARC+ network. Even for a network like NETSARC+, collecting follow‐up data for all patients is an enormous challenge. Despite significant resources, with more than a third of patients not followed up in reference centers, the survey could not be completed for almost 20% of patients [4].

The medico‐administrative database SNDS, “Système National des Données de Santé” (French National Health Data System) [5], is derived from medical care reimbursement data. Universality and exhaustiveness of the data collection are necessary for its primary purpose (reimbursing the insured and participating in the financing of healthcare establishments). Hence, SNDS allows tracking without attrition, as long as the subject remains within the health insurance fold. A date of death is available in SNDS as an issue of a hospital stay or provided by the insurance scheme, but not exhaustively (and sometimes with delay) for other schemes (e.g., agricultural workers and self‐employed workers) than the general scheme. Recently, date and cause of death (analyzed by Inserm Unit CEPIDC from death certificates) are linked to SNDS.

Other highly relevant data, however, would require an unthinkable effort to collect on a large scale, and especially over the long term: All the information needed to describe a patient's course of treatment (consultations, treatments, and hospitalizations). A medico‐administrative database is therefore ideal. On the other hand, data collected for medico‐administrative purposes has limitations (little or no clinical data, no categorization of sarcoma extension at diagnosis, and no histological data). Sarcomas are coded, in the context of hospital stays, by location with organ cancers, which does not enable them to be identified easily, correctly, or exhaustively. Linking clinical data with data already collected systematically for other purposes, such as medico‐administrative data, can overcome data gaps in clinical studies and enable comprehensive analysis of patient care pathways and outcomes [6].

DEEPSARC was one of the first projects running on the Health Data Hub (HDH). One of the challenging aspects of the DEEPSARC project was starting in mid‐2019 in a regulatory and technical context under construction. Established by the Law of 24 July 2019 aiming at the organization and transformation of the French healthcare system, the HDH becomes a public entity that provides a single gateway to assist project leaders with their administrative procedures and a secure, state‐of‐the‐art platform with advanced data storage, computing, and analysis capabilities. The aim of the study was to improve the management of sarcoma patients by identifying real‐life treatment regimens that improve overall survival. The project proposed a nationwide analysis based on matching the national database of the sarcoma reference network (Sarcoma‐BCB) with data from the SNDS, enabling an understanding of patients' care pathways and healthcare consumption over 5 years of follow‐up. The primary objective was to estimate whether the administration of adjuvant and/or neoadjuvant therapy for nonmetastatic sarcomas is associated with better overall survival.

We aimed to report a transparent description of the linking process realized on this platform and its results.

## 2 Material and Methods

### 2.1 Data Collection

On one hand, the Sarcoma‐BCB database allows for describing the population of sarcoma patients in France since 2010. It includes a set of data describing patients and tumor characteristics, surgery, relapse, and survival by cross‐comparison of the clinical database (NETSARC) and the pathological review database for soft tissues and viscera sarcoma (RREPS) and for bone sarcoma (RESOS). A complete description of the three data dictionaries of these three subsets of the database can be found here: https://netsarc.sarcomabcb.org/public/dictionary/display for NETSARC; https://rreps.sarcomabcb.org/public/dictionary/display for RREPS; https://resos.sarcomabcb.org/public/dictionary/display for RESOS.

The selection criteria for the DEEPSARC project in the sarcoma database are a diagnosis of sarcoma according to the 2013 WHO classification of connective tissue tumors, established between January 01, 2010 and December 31, 2017, and surgery of the primary tumor. As the three subsets of the database are not structured to meet the same initial objectives, discrepancies in the data may appear if a patient exists in two of them. These discrepancies are dealt with (using algorithms) by a data manager when linking patients from two subsets in order to prepare the database.

Furthermore, the matching between Sarcoma‐BCB and SNDS is primarily based on the date of surgery, which is the only variable common to both databases. However, in order to provide optimal care for a patient with sarcoma, a biopsy must be performed prior to surgery. It is sometimes impossible or not performed (e.g., when the local team treating the patient suspects a hematoma or cyst rather than sarcoma). The reviewing pathologist confirms the diagnosis of sarcoma based on the first sample received. In the case of a biopsy, only biopsy‐related data are then collected in the database in the pathological side, while surgery‐related data are not, and the surgery procedure is not mentioned. As a result, some patients with pathological data alone are selected without knowing whether they underwent surgery or not (and some are subsequently excluded during the record linkage process when the surgery procedure is not found in the SNDS data).

The French sarcoma reference network NETSARC+ [3], designated by the National Cancer Institute, aims to improve the management of sarcoma patients by ensuring that all of them receive both a definitive diagnosis through double reading of their tumor samples and appropriate treatment through discussion in sarcoma‐dedicated multidisciplinary team meetings (MDTM). A network of sarcoma pathologists (including organ‐specific pathologists) and a network of sarcoma‐specialized MDTM provides these two areas of expertise. NETSARC+ activity is recorded in a dedicated centralized database (Sarcoma‐BCB), with two separate entries allowing the activities of the reviewing pathologists and those of the MDTM to be recorded asynchronously for the same patient. Ideally, each patient should therefore have their data recorded in both sides. Actually, several factors can lead to data not being recorded (e.g., no double reading due to molecular testing confirming the initial diagnosis made in the local hospital, discussion of the case in an organ MDTM instead of a sarcoma MDTM, and the pathologist or sarcoma center not entering their activity exhaustively in the database throughout the year). The Sarcoma‐BCB has recorded around 90% of patients with sarcoma since 2010. The 33 548 patients are divided into three subsets according to the availability of diagnostic and/or clinical data in the database: 13507 patients with a complete dataset gathering clinical and pathological data, which represent 40.3% of the whole population; 5844 patients with clinical data alone, which represent 17.4% of the whole population; 14 197 patients with pathological data alone, which represent 42.3% of the whole population (this latter proportion is significant because it was impossible to select only patients who had undergone surgery). By convention, this database will be called the « source »; the clinical part of it will be called NETSARC while the pathological part of it will be called RREPS (RREPS + RESOS).

On the other hand, the SNDS is built on individual data, linked by pseudonymized record identification from three databases: the SNIIRAM containing health insurance data derived from the processing of healthcare reimbursements, mainly for ambulatory care; the PMSI containing data derived from the healthcare activity delivered in private and public establishments; and the CepiDC database containing data on causes of death derived from the treatment of certificates of death. The SNDS covers around 99% of the French population (which means more than 67 million people) since 2006, and it will allow a follow‐up over a 20‐year sliding window. A comprehensive description of the SNDS database dictionary can be found here https://health‐data‐hub.shinyapps.io/dico‐snds/.

It has been shown that using ICD‐10 code is not a reliable method to identify patients diagnosed with sarcoma due to a lack of consistency in ICD coding for the diagnosis limiting the ability to conduct real‐world observational research of this rare disease [7]. For this reason, the criteria for extracting the subpopulation of the SNDS have been extended to patients who underwent surgery for their cancer between 2010 and 2018, a detailed description is provided in Supporting Information S1: Section A. The CNIL expresses concerns about the size of the subpopulation to be extracted and recommended that the matching step and analysis phase would be carried out in separate environments in order to reduce the risk to expose a 3 million patients' population on the new HDH technological platform. The matching step and analysis phase are indeed carried out in two separate environments, both held by HDH, with restricted grant accesses. By convention, this subset of the SNDS will be called the « target ».

Regulatory approval (Request number 920271) to carry out the linking process is obtained from CNIL (Commission Nationale Informatique et Liberté) on November 20, 2020 (CNIL decision number DR‐202‐360).

### 2.2 Principles of the Record Linkage Process

In order to achieve the objectives of the DEEPSARC project, an initial step of data linkage is needed between the « source » and the « target » databases to enrich the Sarcoma‐BCB database with the complete care pathway of the patients. Data or record linkage has been defined as “a process of pairing records from two files and trying to select the pairs that belong to the same entity [8].” In our case, as no common unique patient identifier is available, an indirect record linkage process is used, retaining several nondirectly identifying variables eventually at hand in the « source » and the « target » databases [9].

The underlying principle is that a combination of such variables (called chaining variables) makes it possible to uniquely identify a patient in each of the bases; such a combination of chaining variables is called a signature (S). However, each chaining variable does not hold the same discriminatory power since, for example, a sex code generally separates all patients into two categories, whereas a year of birth will divide them into groups of smaller sizes.

The algorithm can be described using set theory. For each patient P in the « source » base having a given signature composed of N chaining variables (e.g., sex, birthdate, date of a cancer surgery, and town of residency), it searches among all the patients in the « target » base for the one who has the same signature, or at least the closest subpart of the signature. If a unique patient holding the source signature (or a part of it) can be found in the target, a linked pair has been constituted.

As the algorithm explores the whole research space (i.e., the Cartesian product between patients from the source and target databases), a quality index can be estimated called the robustness (R). The robustness R of a linked pair is the number of chaining variables that can be dropped from the signature without losing the uniqueness of the linked pair. For example, a robustness R = 2 indicates that we could remove any of two variables from the signature without losing the unicity of the link. On the other hand, a robustness *R* = 0 means that there is no possibility of removing any chaining variable from the signature; otherwise, the uniqueness of the link would be lost.

The quality of the information (specifically the rate of missing data for the chaining variables) is obviously a first step.

A software suite (“CUBR‐LINK”, see https://www.cubr.fr/cubr‐link/) has been adapted to the HDH technological platform.

### 2.3 Data Preparation for Record Linkage

#### 2.3.1 Step 1: Application of the Chaining Variables (Six Variables)

In order to prepare the chaining process, a selection of discriminating chaining variables is carried out within the « source » and « target » database. When it is possible, the chaining variables are associated with a date in order to make them more discriminating; for example, a variable C « Type of Tumor », whose modalities are « Soft tissue », « Bone », « Viscera », will be associated with the date of surgery. Furthermore, a margin of error allowed on the dates can be specified in order to resist discrepancies in the process of collecting data in both systems. For example, a variable M « Micro‐biopsy », which represents the presence or absence of a sampling of tumor in the care pathway of the patient, could be associated with the date of the sampling itself in one system and with the date of the production of the result of the tissue fragments in the other database. For the death date, there is no such variable in the « source » database, only a date of last contact that can be combined with a vital status (dead or alive), which means that there could be a variable delay between this combination of variables in the « source » and the real death date in the « target ». For the date of surgery, the « source » database collects the exact date, while in the « target », this information is linked to the beginning of the hospital stay; therefore, the date of surgery could be potentially posterior to it.

The six chaining variables beforehand, available in both target and source databases, are the following: sex, month and year of birth, death date, code of residency (town or department), the hospital where surgery was performed, and the date of surgery. These basic variables are then combined with different levels of granularity (discerned by upper‐ and lower‐case letters): S for sex code + month and year of birth and s for sex code + year of birth, L for code of residency town, and l for code of department of residency (see Table S1 in Supporting Information S1: Section B).

This preparation leads to a « linkage management book » describing the way each chaining variable entering the signature is calculated (see Table S1 in Supporting Information S1: Section B). The details of the code used are in Supporting Information S1: Section B.

At this stage, the chaining process can be launched in order to produce, ideally, a unique linked pair for each patient of the « source » database. In practice, three situations result from the launch of the chaining algorithm: (1) no candidate can be found in the « target » database for a given patient in the « source » database; (2) a unique candidate has been found in the « target » database for a given patient in the « source » database; (3) two or more candidates have been found in the « target » database for a given patient in the « source » database. The matching rate will be calculated by dividing the final number of unique pairs by the total number of patients in the « source » database.

#### 2.3.2 Step 2: Extension to the Checking Variables

If several candidates are eligible for a given patient in the « source » database, one could try to improve the matching rate by integrating more information into the chaining process in order to remove ambiguity among the various candidates in the « target » database. This disambiguation process relies on so‐called checking variables. For example, the NETSARC database collects decisions taken in multidisciplinary team meetings, mainly indications of appropriate treatment options for the patient, like chemotherapy, radiotherapy, or tumor reexcision. Knowing this planned treatment in the near future could help to choose between two candidates by searching for such an event in their care pathway.

Another opportunity is to use diagnosis recorded during the surgery stay, even though ICD‐10 codes were not considered sufficiently reliable at first to identify sarcoma [7]. A limited mapping table between 2000 ICD‐10 codes and a combination of information about the type (Soft tissue, Bone, Viscera) and the anatomical site of the tumor was produced in order to separate the good from the bad when two ambiguous candidates were found after Step1.

The details of the code are in Supporting Information S1: Section C. Table S2 in Supporting Information S1: Section C presents the checking variables used in this step.

However, those variables cannot be used directly as chaining variables since they would have brought too many false positives in the first step of the chaining process, either because the indication for treatment is not necessarily followed by the therapeutic procedure initially planned or because the margin of error on dates on such events could be high or just because the effort to produce a wider mapping table was out of reach.

Finally, a rule of thumb is defined to determine, a priori, which of the patients in the « source » database with several candidates in the « target » database are verified with those checking variables. Two criteria are applied in order to limit this step to patients with a strong concordance with their candidates: (1) the signature of the patient at the end of Step 1 must contain at least five variables, and (2) the signature of the patient at the end of Step 1 must have no more than two missing variables.

The Step 2 is launched for each patient meeting the criteria in the « source » database, the checking variables are compared for each candidate in the « target » database filtered in the Step 1. If a candidate shows at least one checking variable matching with the « source » patient and if no other candidate does, then the pair is considered as potentially unique at the end of Step 2 and improves the matching rate.

#### 2.3.3 Step 3: Elimination of Questionable Pairs

At this stage, all the operations of the algorithm are unsupervised. As stated earlier, sarcoma needs skillful clinician expertise, and the pairing process could not escape the rule. A checking of several care pathways is carried out in order to identify signatures of unique pairs that could seem doubtful to clinicians; the aim is to identify a general rule of safety that automatically eliminates questionable pairs, even if it means accepting the loss of real positives. The criteria are applied to eliminate complete groups of patients with signatures having the following characteristics: The signature does not contain the two chaining variables *S* and *s* (complete lack of demographic information on sex and/or birth date in the « target database »); the signature does not contain the two chaining variables *L* and *l* (complete lack of demographic information on localization in the « target database »); the signature does not contain one out of the two chaining variables *S* or *s*, is missing at least two chaining variables, and contains less than 6 chaining variables; the signature does not contain the chaining variable L, is missing at least two chaining variables, and contains less than eight chaining variables; the signature does not contain one out of the two chaining variables *L* or *l*, is missing at least two chaining variables, and contains less than six chaining variables. The definitive matching rate is calculated at the end of Step 3.

### 2.4 Definition of a Quality Metric

Finally, while robustness can be used to compare individuals within the same signature group, it is less relevant for comparing patients belonging to different signature classes. A metric, called quality (Q), analyzes the results globally, overcoming intraclass variability and enabling inter‐class comparisons of signatures. This metric is defined iteratively as follows: All chained patients at the end of Step 3 are initially assigned a quality of *Q* = 0; *Q* = 1 if pairs present a robustness *R* = 0 with a signature of four or fewer chaining variables; *Q* = 2 if pairs present a robustness *R* = 0 with a signature of more than four chaining variables; *Q* = 3 if pairs present a robustness *R* = 1 with a signature of five or fewer chaining variables; *Q* = 4 if pairs present a robustness *R* = 1 with a signature of more than five chaining variables; *Q* = 5 if pairs present a robustness *R* ≥ 2. In addition, for pairs presenting a quality *Q* ≥ 2: *Q* = *Q* + 1 if at least two checking variables from Step 2 (d, e, c, and r) are present; *Q* = *Q* + 2 if at least three checking variables from Step 2 (d, e, c, and r) are present.

## 3 Results

### 3.1 Evaluation of Missing Data in the Chaining Variables

One of the first results produced was to check the quality of the information and, in particular, the rate of missing data for the expected contributing variables in the pairing process. Table 1 presents the rate of missing values for those variables in the source database, either in the clinical (NETSARC) or pathological subsets (RREPS + RESOS). The demographic variables (sex and birth date) were completely recorded in both subsets since they were needed for creating the hash ID of the patient. However, the geographic origin of the patient was less well recorded in general than for demographic variables, with 15.45% of missing data at the most precise level (town of residency) for the pathological base and 7.59% for the clinical base. The clinical base presented a better filling rate at the different levels; the pathological base alone did not allow for locating the patient at all in 10.74% of cases, even at the regional level. Concerning the variables related to surgery, 170 patients did not have a date of surgery, which is surprising since this was an inclusion criterion in the study. We also observed 230 patients with a missing value for the variable “type of tumor,” which is also surprising since this variable is mandatory and controls the entry of dependent variables in the eCRF. This absence immediately led to the elimination of those patients from any possibility of chaining them to the “target” base. Identification of the surgeon was missing in more than 78% of cases due to the fact that the surgeon's name has only been collected in the sarcoma database since 2016 and only for surgeons in the network. This information can therefore only make a weak contribution to the chaining process and could at best be an element of disambiguation of equivocal pairs.

**TABLE 1:** Rates of missing values for the expected chaining variables in the source database.

Source	Variable	Number of missing values	Percent of missing values (total)
NETSARC	SEX	0	0% (19351)
NETSARC	BIRTH_DATE	0	0% (19351)
NETSARC	GEOGRAPHIC_ORIGIN__INSEE_ (TOWN)	1468	7.59% (19351)
NETSARC	GEOGRAPHIC_ORIGIN__DEPARTMENT_	876	4.53% (19351)
NETSARC	GEOGRAPHIC_ORIGIN__REGION_	326	1.68% (19351)
RREPS	SEX	0	0% (27704)
RREPS	BIRTH_DATE	0	0% (27704)
RREPS	GEOGRAPHIC_ORIGIN__INSEE_ (TOWN)	4281	15.45% (27704)
RREPS	GEOGRAPHIC_ORIGIN__DEPARTMENT_	3360	12.13% (27704)
RREPS	GEOGRAPHIC_ORIGIN__REGION_	2975	10.74% (27704)
NETSARC	SITE_OF_TUMOUR	7	0.04% (19351)
NETSARC	TYPE_OF_TUMOUR	0	0% (19351)
NETSARC	DATE_OF_SURGERY	170	0.88% (19351)
NETSARC	FINESS_GEO_SURGEON_1	15 267	78.90% (19351)
RREPS	TYPE_OF_SAMPLING	1	0.004% (27704)
RREPS	SITE_OF_TUMOUR	8	0.029% (27704)
RREPS	TYPE_OF_TUMOUR	230	0.83% (27704)
RREPS	DATE_OF_SAMPLING	0	0% (27704)

*Note:* NETSARC stands for the sarcoma clinical database, RREPS and RESOS stands for the pathological review database for soft tissues and viscera sarcoma and for bone sarcoma, respectively. In RREPS database, tumor material circulates to review centers, which do not have precise information on the geographical origin of patients.

### 3.2 Results of the Pairing Algorithm

It should be remembered that the pairing algorithm produced three types of results: (1) In the most favorable situation, a unique pair was found between a patient from the source and the target database; (2) in the less favorable situation, there was no combination of chaining variables that could produce a candidate in the target database for a given source patient; (3) in intermediate situations, it could be found a signature that produced several candidates in the target database for a given source patient.

**FIGURE 1:**
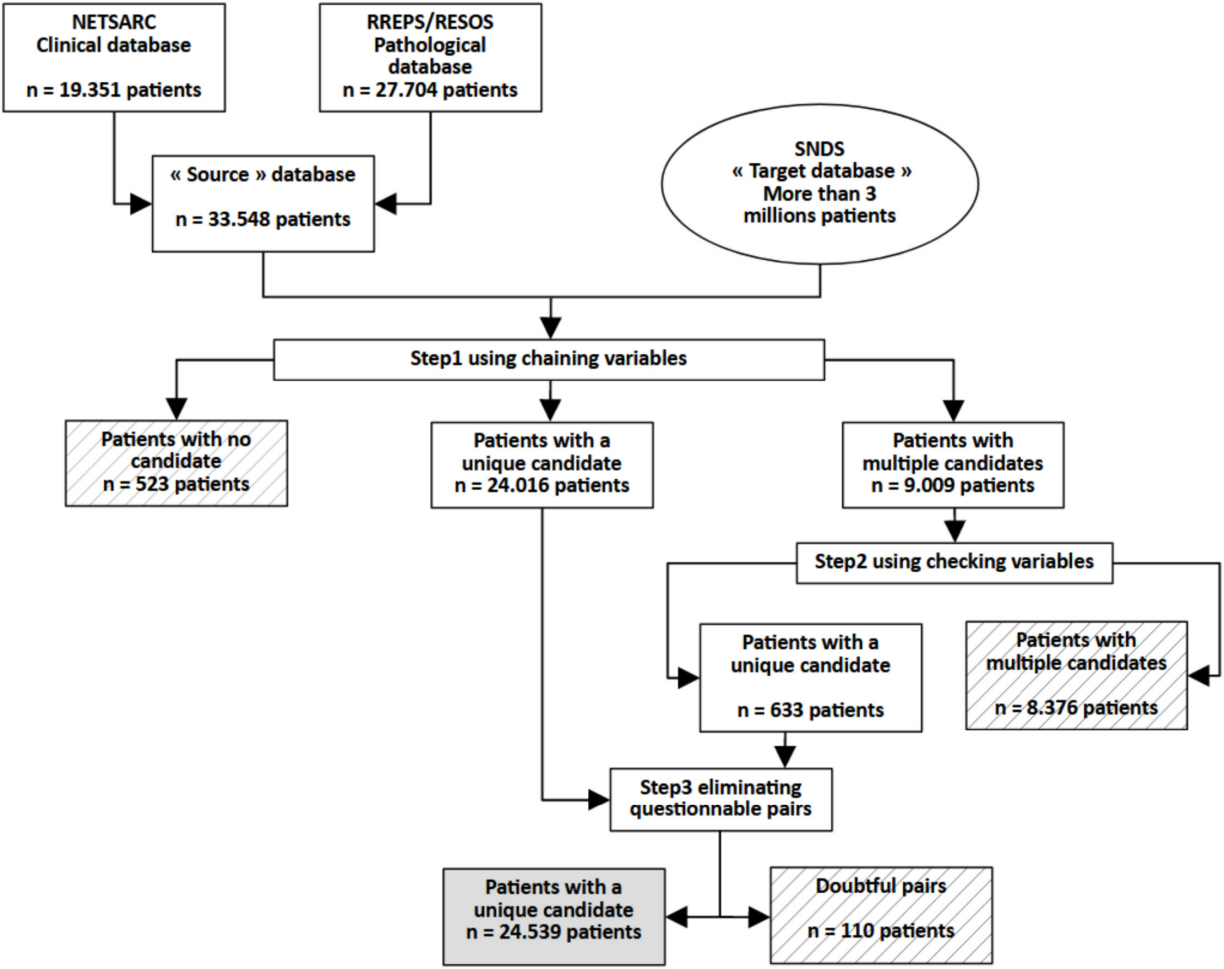
Flowchart of the different steps of the pairing algorithm.

**FIGURE 2:**
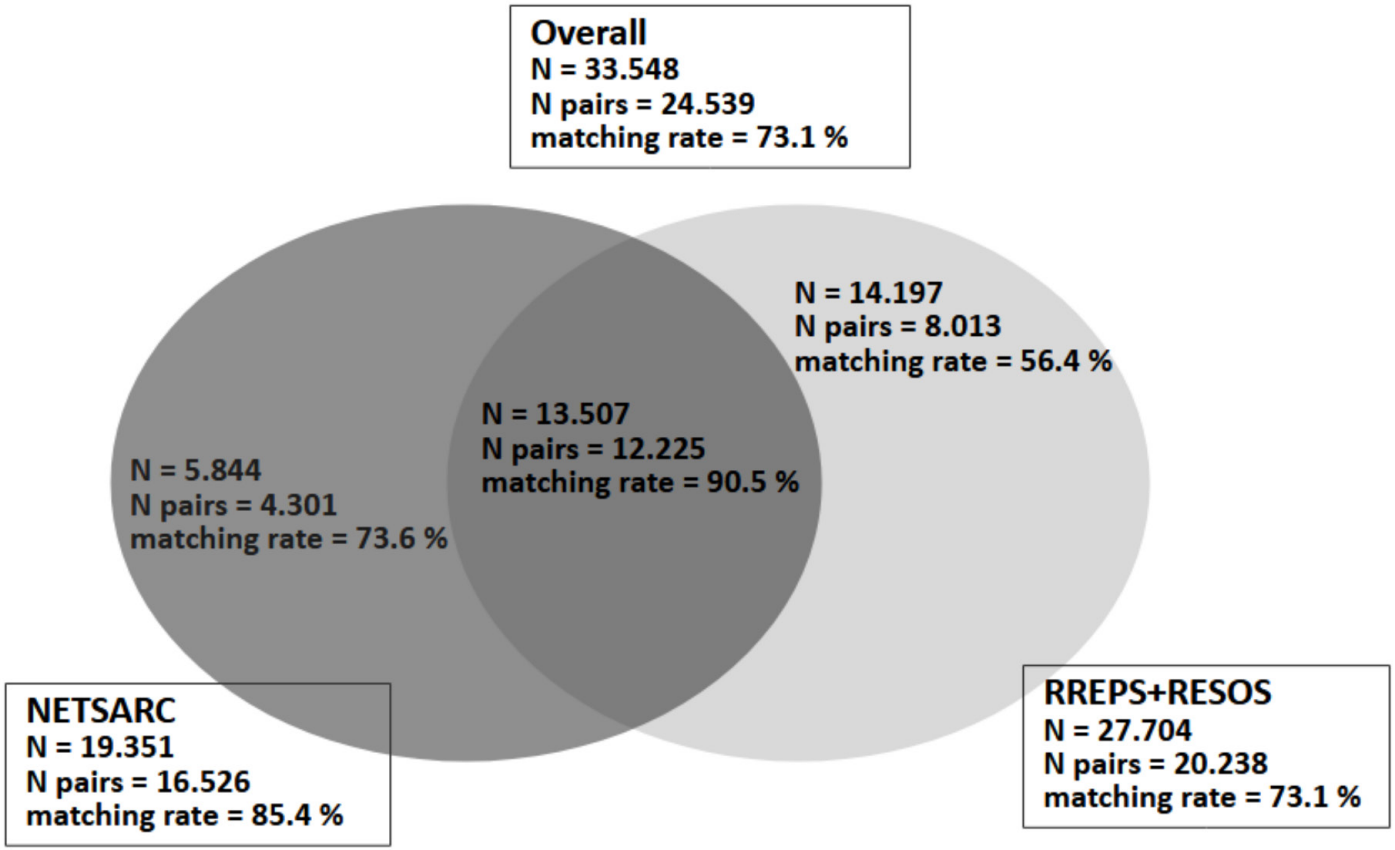
Definitive matching rates of the pairing algorithm by origin of the « source » database. NETSARC stands for the sarcoma clinical database, RREPS and RESOS stands for the pathological review database for soft tissues and viscera sarcoma and for bone sarcoma, respectively.

Figure 1 presents the flowchart of the different steps of the pairing algorithm. Figure 2 gives the definitive matching rates after the three steps of the pairing algorithm according to the origin of the « source » database (NETSARC for clinical data or RREPS + RESOS for pathological data).

Table 2 gives some characteristics of the two subgroups according to the origin of the « source » database with NETSARC for clinical data, RREPS + RESOS for pathological data, INTERSECT for the patients present in both databases, and OVERALL for the whole population.

**TABLE 2:** Characteristics of the paired and nonpaired patients by origin of the « source » database.

Source database	Sex	Characteristic	Paired	Nonpaired
OVERALL	Female	Number (%)	12 336 (74.0%)	4336 (26.0%)
		Age—mean (SD) [min–max]	58.1 (19.7) [0–102]	60.0 (19.8) [0–113]
	Men	Number (%)	12 203 (72.3%)	4673 (27.7%)
		Age—Mean (SD) [min–max]	57.7 (20.8) [0–101]	60.1 (20.7) [0–113]
NETSARC	Female	Number (%)	8362 (85.6%)	1402 (14.4%)
		Age—mean (SD) [min–max]	56.6 (18.9) [0–99]	54.3 (18.9) [0–96]
	Men	Number (%)	8164 (85.1%)	1423 (14.9%)
		Age—mean (SD) [min–max]	56.2 (20.1) [0–99]	54.9 (20.1) [0–96]
RREPS + RESOS	Female	Number (%)	9944 (73.9%)	3510 (26.1%)
		Age—mean (SD) [min–max]	58.6 (19.8) [0–102]	61.6 (19.8) [0–105]
	Men	Number (%)	10 294 (72.2%)	3956 (27.8%)
		Age—mean (SD) [min–max]	58.4 (20.7) [0–101]	61.5 (20.3) [0–113]
INTERSECT	Female	Number (%)	5970 (91.2%)	576 (8.8%)
		Age—mean (SD) [min–max]	56.8 (18.9) [0–97]	55.7 (19.0) [3–93]
	Men	Number (%)	6255 (89.9%)	706 (10.1%)
		Age—mean (SD) [min–max]	57.1 (19.7) [0–99]	57.3 (18.4) [1–96]

*Note:* NETSARC stands for the sarcoma clinical database, RREPS and RESOS stands for the pathological review database for soft tissues and viscera sarcoma and for bone sarcoma, respectively.

The pairing algorithm produced 24 539 unique pairs between the source and the target databases. Those patients are divided into 104 distinct signatures.

A partial view of the results for the 20 first signatures representing almost 89% of the total pairs (*n* = 21 835 pairs) is presented in Table 3, giving for each signature the number of matching pairs and some parameters of the distribution of the robustness variable. The extensive results are available in the Supporting Information S1: Section D. It is finally noticeable that the complete signature (SsDLlCFRMO) is only found for seven patients, which seems to indicate that the algorithm is quite resilient to a partial absence of information since most of the pairs are found with missing chaining variables.

**TABLE 3:**
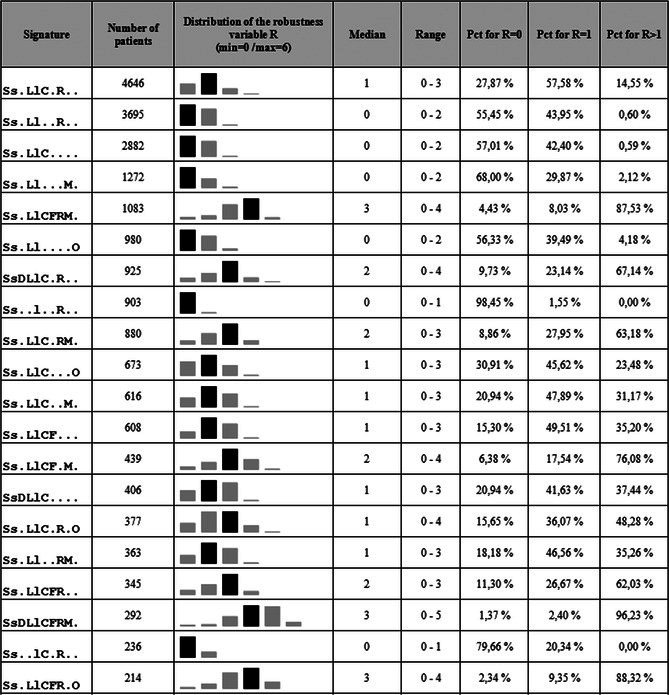
Number of unique matching pairs for the 20 first signatures (*n* = 21 835 patients) with details of the distribution of the robustness variable.

*Note:* The signature “Ss.LlC.R..” allowed matching 4646 patients, meaning that the combination of sex, month and year of birth, town of residency, date of surgery, and date of tumor resection were concordant both in the source and the target. The robustness *R* of a linked pair is the number of chaining variables that can be dropped from the signature without losing the uniqueness of the linked pair. The variable indicating the surgeon who realized the operation was involved in the pairing of 3638 patients, which was a weak overall contribution. However, there was a very high rate of missing values for this variable (n = 15 267 among the 19 351 NETSARC patients). This means that when this value was known, it could be involved as a contributing chaining variable in almost 90% of the cases (3638/(19 351–15 267)).

**FIGURE 3:**
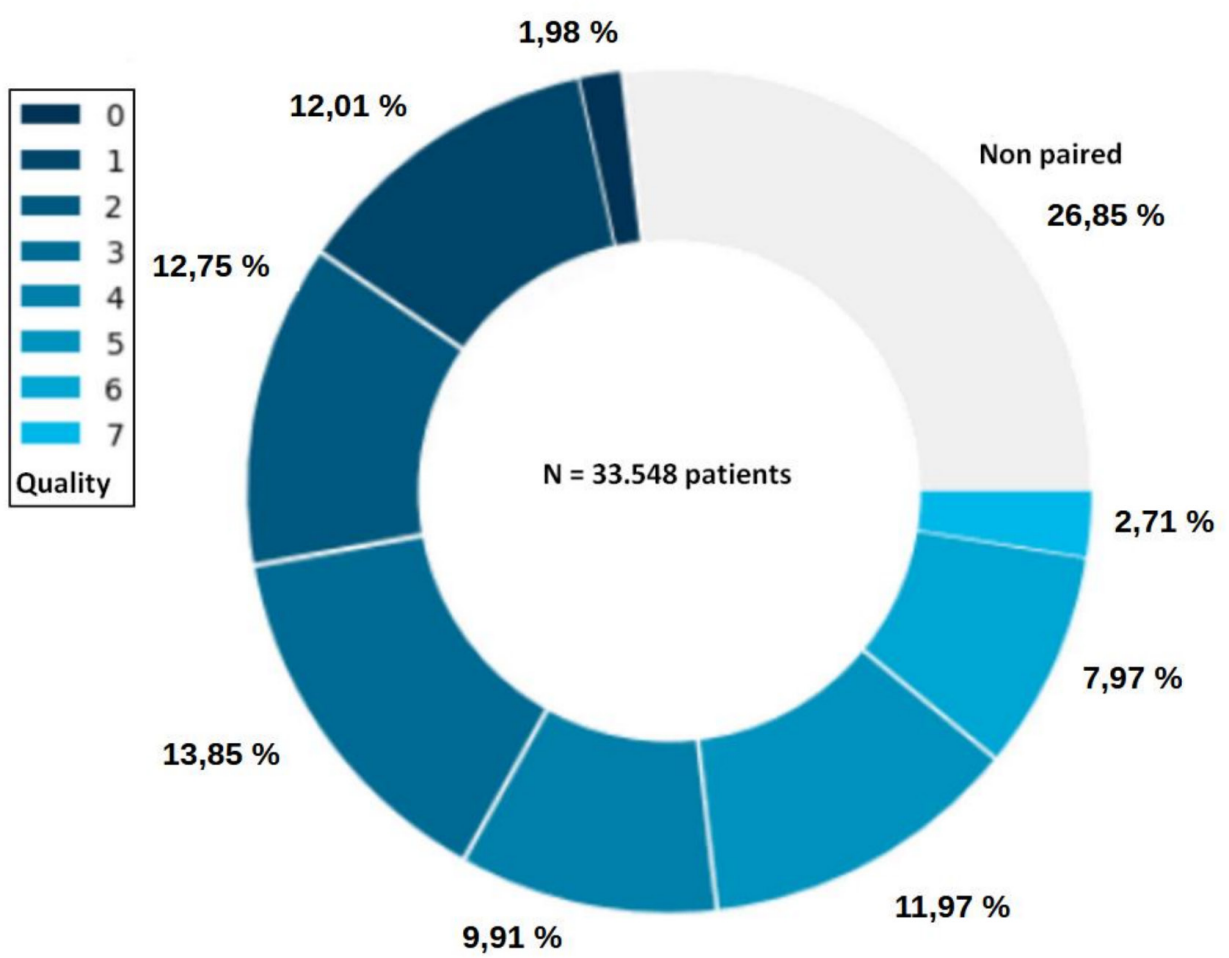
Distribution of the quality metric for the paired patients.

Figure 3 presents the distribution of the quality metric for the paired patients ranging from 0 to 7, the gray sector representing the nonpaired patients (*n* = 9009 patients).

## 4 Discussion

The linking process for the DEEPSARC project faced several challenges: firstly, the heterogeneity of data and completeness, considering a clinical database (NETSARC) and two pathological databases (RREPS and RESOS) on the one hand, and a health insurance database on the other hand; secondly, the large amount of observation, 33 548 patients on the one hand (including 13 507 patients, 40.3%, with extended data, clinical as well as pathological), and 3 million patients who underwent surgery for their cancer between 2010 and 2018, on the other hand.

An indirect, stepwise, combinatory approach (set theory) allowed an overall matching rate of 73.1% (24 539 pairs out of 33 548 observations), reaching 90.5% in the intersection of the sarcomas databases (with extended data, 12 225 pairs out of 13 507 observations). In addition, two metrics, robustness, and quality allowed discussion and interpretation of the linking results.

A number of limitations need to be discussed. The first is that the result of linkage is intimately related to the quality of the data used. In our example, the two databases (NETSARC and RREPS) have a different number of candidate variables, and the quality of the data is also very different, both in terms of typology and discriminative capacity, as well as the percentage of missing data. Logically, not only the linkage rate but also the linkage quality metrics are different, and in the expected direction. This is where the importance of producing these metrics to describe the result of linkage becomes apparent.

A second limitation is the choice of integrating an endpoint (date of death when a survival analysis is planned) as a linking variable: subjects who died within the observation window have a higher probability of being “well” linked, especially as the information will be collected more reliably for individuals who died in hospital, who could therefore be overrepresented among linked subjects. Here again, the metrics delivered, and, in particular, the robustness among decedents provide important elements for the discussion of potential selection bias. Among the 24 539 paired patients, 2613 (10.9%) showed a signature involving the death date. For the very majority of cases (2331 out of 2613, i.e., 89.2%), the robustness of the signatures was greater than 0. This means that the death date in the pairing process brought mainly a reinforcement of the confidence of the uniqueness of the pair but was very marginally the crucial variable to establish a unique pair, since it was not possible to remove this variable in only 282 pairs.

A third limitation is selection bias related to nonlinking. Even a very high linkage rate does not obviate the issue of bias in statistical analysis. It is therefore important to describe and compare linked and unlinked individuals in order to identify any association between the linkage result and the characteristics of the individuals. In our example, an individual present only in the RREPS database has a greater probability of not being linked, and the characteristics of these individuals are different from those of individuals in the NETSARC database, for example, in terms of contact with an expert center for their care.

A final limitation is a bias related to the quality of the pairs constituted, since quality is correlated with the number and availability of linking variables. It is important to be able to conduct sensitivity analyses according to the quality metric.

Several strengths are worth highlighting. First of all, the software suite used can handle databases of several million individuals. This scalability is essential when it comes to linking a clinical database with a medico‐administrative database. Indeed, the combinatorial and agnostic approach compares the signature (combinations of chaining variables) of an individual in the source database with the signatures of all the individuals in the target database. A data organization trick is used to break the computational lock.

Secondly, the software suite is flexible: It allows parameterization of linking variables, such as authorizing a delay between two dates. The speed of processing enables parameters to be adjusted on the fly and results to be compared as a function of this adjustment.

Thirdly, the software is resilient to missing data when dates are available. The first four signatures represent more than 50% of the paired patients (*n* = 12 495), and they only rely on a weak signature with five or six variables among the 10 potential chaining variables—in general, an event date (or 2) along with the demographic variables. This indicates that knowing only a date (surgery date, tumor resection date, or micro‐biopsy date) is very discriminant for the pairing process and, in more than half of the time, even sufficient to find a unique pair. However, those four groups present generally a very low value for robustness (median = 0 when only one date is known, as in SS.LL..R.., SS.LLC. … or SS.LL…M., while median = 1 when two dates are known, as in Ss.LlC.R..), reinforcing the idea that the date variable is essential; otherwise, the uniqueness of the pair is lost.

Finally, the software suite calculates elements for understanding the linking result [9]. This interpretability is based on the production of signatures for linked pairs, signatures for unlinked individuals, and a quality metric. Robustness is a good indicator for comparing pairs within the same signature group. Indeed, the level of confidence in matching a patient with robustness *R* = 2 will be higher than if *R* = 0. However, robustness is less relevant for comparing patients belonging to different signature classes. Indeed, if the signature contains, for example, six chaining variables, obtaining an *R* = 2 robustness for a patient does not have the same significance as for a patient with the same robustness but whose signature contains only three chaining variables. Quality is a metric that can overcome intraclass variability and enable inter‐class comparisons of signatures. The advantage is that it takes into account both the notion of robustness and the context, linked to the information available in the « source » and « target » databases for a given patient, thus smoothing out intersignature variability.

In summary, an optimized and transparent process led to a moderate matching rate but enhanced the confidence in true matching. Representativeness is an issue related to the missing data pattern across the three NETSARC databases. For instance, an individual present only in the RREPS database has a greater probability of not being linked.

Statements relating to ethics and integrity policies: access to the data by an independent expert mandated by a scientific publisher shall be carried out under the conditions set out in Deliberation No. 2018‐155 of May 3, 2018, approving the French Reference Methodology (MR‐004) for the processing of personal data implemented in the context of research not involving human participants, studies, and evaluations in the field of health.

## Author Contributions

Manuscript preparation: E. Drezen, A. Happe, and E. Oger. Concept and design of the linkage process: E. Drezen, A. Happe, and E. Oger. Data production (ICD‐10‐NETSARC mapping): C. Chemin Airiau. DEEPSARC Project Management: H. Crochet and F. Ducimetiere. Linkage software implementation and interpretation: E. Drezen. Critical review: J.Y. Blay, G. Du Bouexic De Pinieux, F. Gouin, F. Le Loarer, S. Mathoulin Pelissier, N. Penel, and V. Thevenet.

Regulatory approval (DR 2020 360) by CNIL on November 20, 2020.

## Funding

NETSARC+ (INCa‐DGOS), RRePS (INCa‐DGOS), RESOS (INCa‐DGOS), INTERSARC+ (INCa), LabEx DEvweCAN (ANR‐10‐LABX‐0061), LYriCAN+ (INCa‐DGOS‐INSERM‐ITMO cancer_18003), Ligue Nationale contre le Cancer, Ligue contre le Cancer (Comité de l'Ain), Fondation ARC, and EURACAN (EU project 739521). The DEEPSARC Project was granted by the Health Data Hub.

## Conflicts of Interest

The authors declare no conflicts of interest.

## Supporting information


**Table S1:** Linkage management book of the DEEPSARC project.
**Table S2:** Checking variables used in Step 2 of the pairing process.

## Data Availability

The data that support the findings of this study are available on request from the corresponding author. The data are not publicly available due to privacy or ethical restrictions.
